# Variations in the Use of Faecal Immunochemical Testing (FIT) in Primary Care in England: A Population-Based Cohort of 531,735 FITs from 495,121 Patients Between 2019 and 2023

**DOI:** 10.2147/CLEP.S518048

**Published:** 2025-06-08

**Authors:** Alastair James Morton, Colin J Crooks, Joe West, Brian D Nicholson, David J Humes

**Affiliations:** 1 School of Medicine, University of Nottingham, Nottingham, UK; 2 National Institute for Health Research (NIHR) Nottingham Biomedical Research Centre (BRC), Nottingham University Hospitals NHS Trust and the University of Nottingham, Nottingham, UK; 3 Nottingham Digestive Diseases Centre, Nottingham University Hospitals NHS Trust, Nottingham, UK; 4 Lifespan and Population Health - School of Medicine, University of Nottingham, Nottingham, UK; 5 Department of Clinical Medicine - Hepatology and Gastroenterology, Aarhus University, Aarhus, Denmark; 6 Nuffield Department of Primary Care Health Sciences, University of Oxford, Oxford, UK

**Keywords:** colorectal cancer, faecal immunochemical testing, health inequalities

## Abstract

**Background/Objectives:**

Faecal Immunochemical Testing (FIT) is recommended for patients presenting to primary care with symptoms suggestive of colorectal cancer. This study quantified variations in use across England.

**Methods:**

Retrospective cohort of English patients (≥18 years) with a FIT result reported in routinely collected primary care records, 2019–2023. Rates of FIT testing by age, sex, year and region were adjusted using Poisson regression. Multivariate logistic regression compared the effect of factors on the proportion of results exceeding the recommended referral threshold (10µgHb/g).

**Results:**

Between 01/01/2019 and 05/06/2023 there were 531,735 FIT results among 495,121 patients. Rates of testing increased from 0.69 per thousand person-years in 2019 (95% CI 0.68–0.71) to 27.70 in 2023 (95% CI 27.56–27.85). There were large variations in testing between regions, with rates >3-fold higher in the Northeast than the West Midlands: 17.05 (95% CI 16.87–17.23) versus 4.72 (95% CI 4.67–4.76) per thousand person-years. About 20.4% of FIT results were ≥10µgHb/g. Despite increased testing, this did not change over time. The proportion of FIT ≥10µgHb/g was lower in regions with higher rates of testing, from 16.7% (Southwest) to 25.3% (Southeast; rates of testing 14.62 and 8.00 per thousand person-years respectively). This difference in proportion of FIT ≥10µgHb/g persisted after adjusting for year, sex and age (OR 0.57, 95% CI 0.55–0.58).

**Conclusion:**

Rapid increases in FIT testing in primary care show large, persistent variations between English regions, which correlate with the proportion of results meeting the criteria for onward referral. Differences in the population tested and FIT’s implementation between regions are likely to explain these variations.

## Plain Language Summary

**1) Why was this study done?**

- A simple test for blood in the poo, called FIT, can help decide whether a person with bowel symptoms seeing their primary care doctor should be referred to a hospital for further bowel investigations.
- Currently, a FIT result of 10 µgHb/g faeces or more is recommended to guide referral for bowel tests.
- No study has reported how FIT use, or results, differs across England.

**2) What did the researchers find?**

- This is the first study on national patterns of FIT testing, showing a rapid but unequal increase across England.
- There is large variation in the proportion of FIT results exceeding the referral criteria of 10 µgHb/g faeces between English regions that is associated with the rate of testing in each region.

**3) What do these results mean?**

- Using a single FIT value as a threshold for onwards referral may work differently in different regions of England. As FIT is adopted more widely and rates of testing increase, primary care services may see a decrease in the proportion of patients with a FIT result reported over the referral threshold.

## Introduction

The global burden of colorectal cancer (CRC) is increasing.^1^ In the UK, 42,000 people are diagnosed a year with just 10% diagnosed within the screening programme.^2^,^3^ Cancer referral pathways have been based on symptoms that are poor at predicting the risk of CRC.^4^,^5^ This makes it difficult to identify patients in primary care at risk of CRC to refer on for investigation.

Faecal Immunochemical Testing (FIT) is a non-invasive test that can be performed at home, detecting small amounts of blood in the stool. Identifying asymptomatic patients through the use of FIT in screening is associated with reductions in the incidence of advanced-stage CRC and mortality.^6^ FIT was subsequently recommended to guide referral of patients with symptoms suggestive of CRC by the National Institute of Health and Care Excellence (NICE) from 2017 (updated 2023),^7^,^8^ and specialty bodies in 2022.^9^ Implementation of NICE guidance has varied by NHS region, with hospital centres and commissioning bodies developing their own FIT pathways.^10–13^ Published studies using local data, whilst from pioneering centres,^10–13^ may not be representative of the national picture. As FIT now represents a gateway “triage” test to referral, understanding variations in FIT use is key to understanding the impact of regional differences in FIT implementation and a necessary step to achieving equitable access to further investigation and diagnosis of colorectal cancer.

This study aims to use routinely collected primary care records to compare the use of FIT and proportion meeting the threshold for referral over time and between regions.

## Methods

### Data Source

We used routinely collected primary care electronic healthcare records within the Clinical Practice Research Datalink (CPRD) Aurum. CPRD Aurum is a large, population-based dataset of routinely collected, anonymised primary care records for use in clinical research, collected from practices using the EMIS Web^®^ healthcare records system. It contains data from 60 million patients from over 2,000 practices across the UK, of which 18 million patients are alive and currently contributing to the data (>25% population),^14^ and is representative of the national population.^15^ Tests in primary care (such as FIT) are recorded as observations within CPRD Aurum, alongside additional information such as date, result value and units.^15^ CPRD Aurum has been used extensively to describe observations and test results previously.^16–19^

### Study Population

We identified all codes related to FIT use in adults (≥18 years old) recorded in CPRD Aurum from 01/01/2015 to 05/06/2023 using a broad code list (Appendix 1). Due to minimal use of FIT in the primary care population prior to 2019, 01/01/2019 was chosen as the study start date. A patient’s first FIT code with a result each calendar year was used for primary analysis. Any FIT coding that occurred outside of patients’ research acceptable period was excluded (prior to study start date or patient registration at practice, or after registration end date, death date or practice last collection date), or from patient records not marked by CPRD as up to standard for research. Some codes relating to FIT were administrative, eg, FIT kits being sent out, FIT kits not received back, or FIT not completed so no result recorded. In addition, codes related to the faecal occult blood (FOB) tests that had preceded FIT might be misused. Therefore, to capture all FIT related activity, any coding related to FIT was extracted, including these administrative codes and any overlap with FOB testing. Whilst our primary analysis focused on returned FITs with a recorded numerical result, we also assessed if any observed variations were related to the process or coding by performing a sensitivity analysis including all codes related to administration, and FIT or FOB codes without a returned value.

### Denominator Population

The population at risk was generated from all “acceptable” patients in the September 2023 release of CPRD Aurum.^20^ Patients who only had data from prior to the study start date (01/01/2019) were recorded as over 110 years old or had an end date (registration end, death date or practice last collection date) prior to their practice registration start date were excluded.

To generate a denominator for each calendar year, patients were included if they contributed any data to CPRD in that calendar year. Any patients <18 years old in that calendar year were excluded. Age was generated for that calendar year from the year of birth using a date of the 1st January. For each patient, a person-time contribution per year was generated using the latest of the 1st January or their registration date as their start date, and the earliest of 31st December, death date, registration end date or practice last collection date as their end date. Patients who were included in the study population were censored from the denominator population on the date of their first FIT result each calendar year.

### Exposure and Outcome Definition

Age was calculated from year of birth for each calendar year in the study and denominator populations. Age was divided into 10-year bands for those ≥40 years (<40 and ≥90 years were included as separate categories) and then collapsed to 3 categories (18–59, 60–74 and 75+ years) for regression analysis.^21^ Sex was directly coded within CPRD. Due to very small numbers, patients with indeterminate sex were excluded to avoid risk of identification. Region is defined in CPRD as one of the 9 Office of National Statistics (ONS) regions of England.^15^ Wales, Scotland and Northern Ireland were not included as they did not contribute data towards CPRD Aurum throughout the study period. The reference region was defined as the Southeast as it had the largest CPRD population. Year was defined as calendar year from 1st January to 31st December inclusive. The outcome was the first recorded FIT result within each calendar year.

### Statistical Analysis

#### Rates of FIT Testing

To describe FIT testing in each group and region, the total number of FITs undertaken each year were described for sex, age groups, year and region. Rates of testing per 1,000 person years were calculated for each of these groups based on the person-years in the CPRD population denominator per group each calendar year. Rates were plotted for each 10-year age band by year. Rates of FIT testing between regions were plotted over time and mapped on to a geographical map of England using the ONS Open Geography shapefile (https://geoportal.statistics.gov.uk/). The proportion of patients having a repeat FIT in subsequent years was reported by region.

#### Poisson Regression

To assess whether sex, age, year or region have an effect on FIT testing, Poisson regression was used to estimate Incidence Rate Ratios (IRR) and their confidence intervals for rates of FIT testing compared to a reference group, first via a univariate analysis of the effect of the exposure variables: sex, age (3 groups), year of test and region and then a multivariate model with all variables fitted. To assess how differences in testing between regions was related to the other exposure variables, the 2-way interaction between each of these exposure variables was assessed and each variable fitted into the final adjusted, multivariate Poisson model. This generated IRRs for each exposure variable for rates of FIT testing, mutually adjusted for all other exposure variables.

#### Sensitivity Analysis

To explore whether differences in rates of FIT testing were explained by differences in the FIT process recording or increased rates of screening, two further cohorts were made for sensitivity analysis: 1) all codes related to FIT outside of screening (including administrative codes and FOB or FIT codes without a result) and 2) tests coded as screening within CPRD. Both these cohorts were analysed by year in the same manner as the primary analysis, taking only the first recorded observation per patient per calendar year.

#### Distribution of FIT Values

The distributions of the reported FIT results were explored using histograms and high-frequency modes. The recommended threshold for referral was ≥10 µgHb/g faeces based on the latest NICE and specialty guidelines for secondary care referral.^8^,^9^ Comparisons were therefore made using a threshold of ≥10 µgHb/g faeces. This allowed an estimation of which patients would be likely to be referred for further investigation. Results within CPRD were stored as numerical variables, so were missing any comparator symbols (such as “<” or “>”). Published analysis of GP records shows that the most common lower bounds for FIT results are 2, 4, 7 and 10 µgHb/g faeces, with >90–95% of results at these values associated with a “<” or “≤” comparator symbol.^22^ This had implications on results recorded as exactly 10 µgHb/g faeces, as it is likely the vast majority of these were missing the expected “<” symbol due to how this variable was coded within CPRD (and therefore represent results below the NICE referral threshold of ≥10 µgHb/g faeces). Therefore, a validation of results of 10 µgHb/g faeces was undertaken in an external dataset with comparators available, which showed that only 0.18% of values were reported as exactly 10 µgHb/g faeces (Appendix 2). Therefore, for this analysis, results of 10 µgHb/g faeces were considered to be missing the “<” comparator and treated as sub-threshold, with results above 10 µgHb/g faeces henceforth referred to as “FIT10”.

To determine if the proportion of tests that would trigger onward referral according to current NICE guidance changed across different exposures, the proportion FIT10 were compared over time, and between age groups, sexes and regions using descriptive statistics. A univariate logistic regression model was constructed to assess the difference in FIT10 by sex, age, year and region, then an adjusted model was used to account for the effect of each of these other variables. The proportion FIT10 was plotted against the rate of testing per thousand person-years in each region. A Spearman’s rank correlation coefficient was calculated to describe this relationship. The proportion FIT10 was also plotted in each region over time.

All statistical analysis was completed using RStudio 2023.06.1 Build 524 (©2009–2023 Posit Software, PBC). This study was approved by CPRD’s Research Data Governance (RDG) Process (protocol #23_002720; https://www.cprd.com/approved-studies/health-inequalities-and-impact-faecal-immunochemical-testing-fit-symptomatic).

#### Patient and Public Involvement

A diverse group of patients and carers of those with bowel cancer, or other bowel conditions, was engaged in planning this study. This group was formed from participants from different regions across the country. Input from this group has been used to develop the research question based on what is important for patients; the research plan has been reviewed and iterated by the group across multiple meetings. The group will review and contribute to plain-language materials sent out to clinicians and the public highlighting the findings from the study.

## Results

There were 864,037 FIT observations from 672,277 patients, after excluding duplicates and screening tests. 557,337 had a result recorded, and after excluding 25,602 repeated tests within the same calendar year from 24,053 patients (4.9% of patients), the final study population comprised 531,735 FIT results from 495,121 patients (Supplementary Figure 1). This population included 34,031 patients (6.9%) who had at least one further FIT in a subsequent calendar year.

17,910,681 unique patients in CPRD were registered to a practice within the study period, forming the denominator population. Distribution of FIT by sex, age group, year and region are displayed in Tables 1 and 2.

**Table 1:** Rates of FIT Testing in England 2019–2023 by Sex, Age, Year and Region, with Poisson Regression (531,735 Tests)

Demographic	Person Years in CPRD Population	Rate per Thousand PY (95% CI)	Unadjusted IRR (95% CI)	Adjusted IRR^a^ (95% CI)
Male	27049120	8.44 (8.40, 8.47)	ref	ref
Female	26668164	11.38 (11.34, 11.42)	1.35 (1.34, 1.36)	1.27 (1.26, 1.28)
Age 18–59	38592703	5.76 (5.73, 5.78)	ref	ref
60–74	9601349	16.54 (16.46, 16.62)	2.87 (2.85, 2.89)	3.05 (3.03, 3.07)
75+	5523231	27.31 (27.17, 27.44)	4.74 (4.71, 4.78)	4.97 (4.94, 5.01)
Year 2019	12080584	0.69 (0.68, 0.71)	ref	ref
2020	12127424	3.06 (3.03, 3.09)	4.41 (4.31, 4.51)	4.42 (4.32, 4.53)
2021	12228672	8.68 (8.63, 8.74)	12.50 (12.22, 12.78)	12.54 (12.27, 12.82)
2022	12144211	19.57 (19.50, 19.65)	28.17 (27.56, 28.79)	28.31 (27.70, 28.93)
2023	5136390	27.70 (27.56, 27.85)	39.87 (39.00, 40.76)	39.67 (38.81, 40.56)
South East	11735004	8.00 (7.95, 8.06)	ref	ref
North East	1998207	17.05 (16.87, 17.23)	2.13 (2.10, 2.16)	2.15 (2.13, 2.18)
North West	10041855	7.83 (7.77, 7.88)	0.98 (0.97, 0.99)	0.97 (0.96, 0.98)
Yorkshire and The Humber	1686228	9.11 (8.97, 9.26)	1.14 (1.12, 1.16)	1.29 (1.26, 1.31)
East Midlands	1240452	7.15 (7.00, 7.30)	0.89 (0.87, 0.91)	1.04 (1.01, 1.06)
West Midlands	8639663	4.72 (4.67, 4.76)	0.59 (0.58, 0.60)	0.57 (0.57, 0.58)
East of England	1946985	16.26 (16.08, 16.44)	2.03 (2.01, 2.06)	1.99 (1.97, 2.02)
London	10911799	13.55 (13.48, 13.62)	1.69 (1.68, 1.71)	2.01 (1.99, 2.03)
South West	5517087	14.62 (14.52, 14.73)	1.83 (1.81, 1.84)	1.82 (1.81, 1.84)

**Notes**: Includes all SNOMEDCT codes with a numerical FIT result. ^a^Mutually adjusted by for all other variables in the table.

**Abbreviations**: PY, person-years; IRR, Incidence Rate Ratio.

**Table 2:** The Number and Proportion of FIT Results ≥10 µgHb/g Faeces (2019–2023)

Demographic	Total Tests	Number FIT10 (%)			Unadjusted OR FIT10 (95% CI)		Adjusted OR FIT10 (95% CI)	
Male	228177	51511		(22.58)	ref		ref	
Female	303558	56892		(18.74)	0.79	(0.78–0.80)	0.81	(0.80 – 0.82)
Age 18–39	53044	8049		(15.17)	ref		ref	
40–49	64844	9055		(13.96)	0.91	(0.88 – 0.94)	0.91	(0.88 – 0.94)
50–59	104248	16107		(15.45)	1.02	(0.99 – 1.05)	1.02	(0.99 – 1.05)
60–69	103041	19139		(18.57)	1.28	(1.24 – 1.31)	1.26	(1.22 – 1.30)
70–79	117653	27721		(23.56)	1.72	(1.68 – 1.77)	1.69	(1.64 – 1.74)
80–89	76065	23487		(30.88)	2.50	(2.43 – 2.57)	2.44	(2.38 – 2.51)
90+	12,840	4845		(37.73)	3.39	(3.25 – 3.54)	3.33	(3.19 – 3.47)
Year 2019	8395	1500		(17.87)	ref		ref	
2020	37151	8346		(22.47)	1.33	(1.25 – 1.42)	1.30	(1.23 – 1.39)
2021	106185	22035		(20.75)	1.2	(1.14 – 1.28)	1.14	(1.07 – 1.21)
2022	237708	48373		(20.35)	1.17	(1.11 – 1.24)	1.04	(0.98 – 1.10)
2023	142296	28,149		(19.78)	1.13	(1.07 – 1.20)	0.98	(0.93 – 1.04)
South East	93931	23783		(25.32)	ref		ref	
North East	34070	6346		(18.63)	0.68	(0.65 – 0.70)	0.67	(0.65 – 0.69)
North West	78584	17234		(21.93)	0.83	(0.81 – 0.85)	0.85	(0.83 – 0.87)
Yorkshire & The Humber	15367	3001		(19.53)	0.72	(0.69 – 0.75)	0.71	(0.68 – 0.74)
East Midlands	8870	2116		(23.86)	0.92	(0.88 – 0.97)	0.89	(0.85 – 0.94)
West Midlands	40753	9557		(23.45)	0.9	(0.88 – 0.93)	0.91	(0.88 – 0.94)
East of England	31665	6220		(19.64)	0.72	(0.70 – 0.74)	0.70	(0.68 – 0.73)
London	147814	26682		(18.05)	0.65	(0.64 – 0.66)	0.68	(0.67 – 0.69)
South West	80681	13464		(16.69)	0.59	(0.58 – 0.60)	0.57	(0.55 – 0.58)

**Abbreviations**: OR, odds ratio; FIT10, tests ≥10 µgHb/g faeces.

### FIT Testing Over Time, by Sex and Age

FIT testing increased over time, from 8,395 tests in 2019 to 237,708 tests in 2022 (the last full year of data). Rates of testing increased 40-fold from 0.69 tests per 1000 person-years in 2019 (95% CI 0.68–0.71) to 27.70 per thousand person-years in 2023 (95% CI 27.56–27.85, Table 1). Women completed more FITs than men; 11.38 (95% CI 11.34–11.42) compared to 8.44 (95% CI 8.40–8.47) per thousand person-years (IRR 1.35, 95% CI 1.34–1.36). The rate of testing increased with age, from 5.76 (95% CI 5.73–5.78) per thousand person-years in those 18–59 years, 16.54 (95% CI 16.46–16.62) in those 60–74 years to 27.31 (95% CI 27.17–27.44) in those 75+ years (Table 1). Across England, the rate of testing in over 75s in 2023 was 74.3 per 1000 person-years.

### FIT Testing by Region

**Figure 1:**
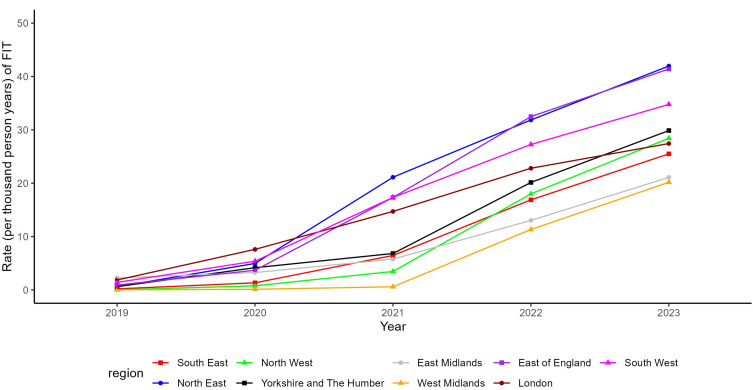
Rates of FIT testing over time between regions of England (2019–2023).

There was a wide variation in FIT testing between regions, with the highest rates of testing in the Northeast and lowest rates in the West Midlands (17.05 per thousand person-years, 95% CI 16.87–17.23 versus 4.72 per thousand person-years, 95% CI 4.67–4.76). Figure 1 shows how testing varies by region over time (mapped in Supplementary Figure 2). Regions with lower rates of FIT testing in 2019 were lower through the entire study period, for example, the West Midlands 2023 rates were 20.17 per thousand person-years compared to over 40 in both the Northeast and East of England. Exceptions to this were seen in both the East Midlands and London, which started with the highest rates of FIT testing in 2019 (2.24 and 1.86 per thousand person-years respectively) but had a later increase compared to the rest of the country (21.09 and 27.43 per thousand person-years in 2023). The region with the highest proportion of patients having a repeat FIT in subsequent years was London (10.3%), and the lowest was the West Midlands (2.37%).

### Adjusted Poisson Model

Incidence Rate Ratios for use of FIT were mutually adjusted in the Poisson model (Table 1). There was minimal effect on IRRs, with the trends described above remaining once adjusted for age, sex, calendar year and region. There was also minimal change in the IRRs when modelling year as a continuous or categorical variable. When modelled continuously, FIT testing doubled each year (IRR 2.12, 95% CI 2.12–2.13).

### Sensitivity Analysis

To assess whether variations in FIT testing were explained by poor return or recording of results in certain regions, the analysis was repeated to also include all non-screening FIT codes without a result (739,802 observations from 672,277 patients). Including tests without a result did not have a major impact on the observed regional variation (Supplementary Figure 3). The only exception was in the West Midlands, where only 55% of FITs had a result compared to 91% in the East of England (Supplementary Table 1). Including these FIT codes without a result in the analysis, as evidence of FIT-related testing, showed testing increased earlier in the West Midlands than in the primary analysis, with an increased rate of testing observed from 2021.

Finally, a sensitivity analysis was conducted to look at screening tests, to explore if areas with lower FIT testing were offset by using more screening tests. A total of 5,518,555 screening tests from 3,439,420 patients within CPRD were assessed. 136,794 of these tests were related to bowel scope screening tests (flexible sigmoidoscopy screening). The majority of screening tests were in those eligible for the screening programme aged 60–74 years, with rates unchanged over time apart from an expected dip during the COVID-19 pandemic in 2020. When adjusted for age, sex and year, there were minimal differences in screening rates between regions (Supplementary Table 2), with similar rates between regions each calendar year within the screening age range (Supplementary Figure 4).

### Distribution of FIT Values

The most frequently reported lower value mode was 4 µgHb/g faeces (109,669 results), followed by 10, 7 and 2 µgHb/g faeces (74,285, 69,411 and 68,734 results, respectively), with higher value modes 200 and 400 µgHb/g faeces (10,117 and 7,924 results, Supplementary Figure 5).

**Figure 2:**
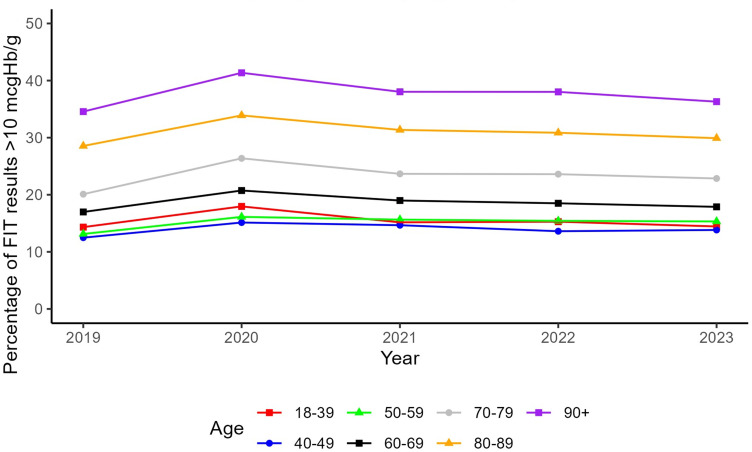
Percentage of FIT results ≥10 μgHb/g faeces (FIT10), by age group and year.

108,403 of results were FIT10 (20.4%). Table 2 shows how the proportion FIT10 varies by sex, age, year and region. Men had a higher proportion FIT10 than women: 51,511 of 228,177 (22.6%) compared to 56,892 of 303,558 (18.7%). FIT10 increased with age from 60 years, from ~15% below 60 up to 37.7% in those over 90 years old (Table 2, Supplementary Figure 6). The proportion FIT10 stayed consistent over time across all age groups, despite an increase in testing, with the exception of a national increase in 2020 (Figure 2).

**Figure 3:**
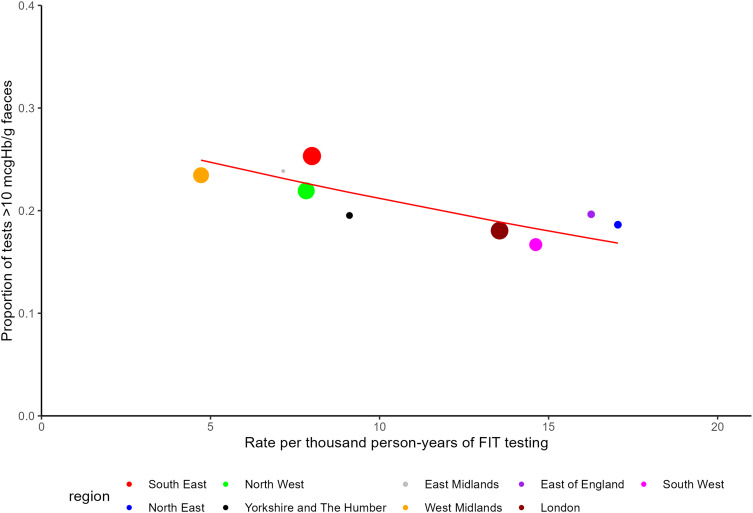
Variation in FIT results ≥10 μgHb/g faeces (FIT10) by regional rates of FIT testing. Size of data points corresponds to number of person-years within each region denominator. Spearman’s rank correlation coefficient rho = −0.70. Regression line predicted from a generalised linear model, weighted by the person years in the CPRD denominator for each region, was drawn to display the relationship between proportions FIT10 and the rate of testing in different regions.

There was a large difference in the proportion of FIT10 between regions, from 16.7% in the Southwest to 25.3% in the Southeast (unadjusted logistic regression OR 0.59, 95% CI 0.58–0.60). This difference in FIT10 between regions was not explained by regional differences in sex, age or the year of testing when adjusted for these in the logistic regression model (Table 2). The proportion FIT10 between regions was correlated to the rates of testing within these regions, with decreasing proportions FIT10 in regions with higher rates of testing (Spearman’s rank correlation coefficient 0.70, Figure 3). The difference between regions showed some convergence over time, from a range of 16.6–26.7% (10.1%) in 2021 (some regions had too few tests prior to 2021 to draw meaningful conclusions) to 15.8–23.7% (7.9%) in 2023 (Supplementary Figure 7).

## Discussion

### Key Findings

We found that the use of FIT testing in primary care has rapidly increased since 2019 in England, with rates doubling every year. This rapid increase varied substantially across the country with variation in testing persisting over time. Regions slow to use FIT at first were still testing less than other regions at the end of the study period. We found that the proportion of FIT results ≥10 µgHb/g faeces (FIT10) also demonstrated large regional variation linked to the rate of testing in each region. We observed a lower proportion of FIT10 in regions with higher rates of testing. Unmeasured population characteristics and differences in the implementation of FIT between English National Health Service regions is likely to explain the variations we have observed in FIT10. Differences in patient awareness of and behaviour in response to symptoms of possible colorectal cancer, variation in patient access to primary care and FIT testing, underlying sociodemographic and comorbidity patterns of the populations tested, and local policy decisions on accessing testing and referral within primary care, secondary care trusts, or Integrated Care Systems could all contribute to these variations.

### Results in Context of Other Work

Although the use of FIT was first included in national guidance for urgent colorectal cancer diagnostic pathways in 2017,^7^ the rapid adoption of FIT after 2019 relates to the COVID-19 pandemic. This was driven by NHS England advising use of FIT to triage endoscopy during the pandemic to manage the increased waiting times for investigation after suspension of endoscopy services.^23^,^24^ Further drivers for the increase were the introduction of national guidance on FIT testing in 2022 and 2023 from specialty bodies and NICE.^8^,^9^

There is no published literature to date on the variations in national FIT use for patients with symptoms. The FIT results we observed were consistent with the distribution in the literature, with the most commonly reported values (2, 4, 7, 10, 200, 400 µgHb/g faeces) aligning with the reported lower and upper limits from different analysers and published primary care data.^22^,^25–27^ The percentage exceeding the NICE referral threshold of 10 µgHb/g faeces (FIT10), 20.4%, was consistent with the published literature from regional studies^13^,^28^ and a recent National Health Service (NHS) England communication.^29^ Rates of repeat FIT testing were lower, at 4.9% within the year and 6.9% in any subsequent year, than the 9.1% reported in Scotland.^30^

The range of regional variations in FIT10 within CPRD from 16.7% (Southwest) to 25.3% (Southeast) is in keeping with the range reported in a systematic review by Saw et al.^31^ However, this systematic review only included studies with data from prior to March 2020. By definition, this review included minimal data on the change in practice and patient populations tested that occurred during the COVID-19 pandemic, also predating the NICE and specialty body guidance and subsequent exponential rise in FIT we have demonstrated in the contemporary data in this study. It included studies across multiple countries, healthcare systems, patient symptom groups and FIT analysers, which may not be applicable to the structure and implementation of FIT within the NHS. Therefore, our data presents a more contemporary estimate of the proportion of patients who may be considered for secondary care referral for suspected cancer in the English primary care population. This contemporary data is important for informing future policy decisions around the use of FIT.

Published regional studies report a range of FIT10 proportions including 21.9% in Dundee (up to 2016),^32^ 20.7% in Nottingham (up to 2019),^28^ 19% across 50 English hospitals using FIT in a secondary care setting in the NICEFIT study (also only including data up to 2019),^33^ 16% in London (2017)^13^ and as low as 10% in Oxfordshire (up to March 2020).^11^ These FIT studies also predate the NICE and specialty guidance, COVID-19 pandemic and large-scale increase in testing. Our study captures data from after these events that have impacted FIT use. The population included in our analysis provides an overview of testing across England by combining data from multiple regional populations and subregional subpopulations each with unique approaches to FIT testing and with varied sociodemographic profiles with characteristics affecting the FIT results and associated cancer risk.

### Clinical Significance

Despite publication of national guidance, it is likely that local or regional structural differences in the use or implementation of FIT in symptomatic pathways may explain the variation in FIT testing and the results that we have observed in this national study. Implementation details of FIT pathways are decided regionally within the NHS Integrated Care Boards (previously Clinical Commissioning Groups), which may have differing priorities and therefore advocate different referral pathways, but these details are not available nationwide. Differing regional priorities could consequently lead to delayed implementation of guidance or reduced access to FIT testing, resulting in lower rates of testing. In secondary care, pathways for referral from primary care may differ in whether FIT is encouraged or not. For example, institutions that have pioneered the use of FIT may drive rapid uptake in their respective regions, where test facilities and referral pathways are already established. Finally, there may be local variation in use within primary care, either between different general practitioners or primary care practices, and population differences between regions that we have not been able to account for. National guidance from NICE and specialty bodies has attempted to standardise pathways from the top down,^7–9^ but it is clear from our study that variations in testing persist between regions despite this, which may have large implications on resource allocation.

### Strengths and Limitations

This is the first population-based study reporting the patterns of use of FIT in symptomatic pathways across England, since it was recommended in a stand-alone national NICE guideline in 2017.^7^ A key strength is the large numbers and representativeness of the English population in this study. Previous studies have focussed on the use of FIT within established local pathways, whereas our study presents data on use across a nationally representative sample of England, showing large variations between regions that were not demonstrated in local studies. Our study is therefore able to give a picture of the anticipated burden of rates of testing from symptomatic presentation in primary care, the associated laboratory demand, and the potential resulting secondary care referrals.

Our sensitivity analysis included FIT observations without a result, to assess if we were underestimating rates of testing due to differences in coding of data. Broadly, there was very little change from including administrative codes and potentially incomplete tests without results, except for the West Midlands where more administrative codes were used.

Unfortunately, we were not able to assess the impact of ethnicity and deprivation on FIT use. This was because these data were only available up to early 2021 at the time of our analysis so were not contemporary enough for the vast majority of patients completing FITs in our study. This is an area planned for future work when the data allows, as these sociodemographic patient and population factors could explain some of the regional variation observed. Details on the type of analyser used for processing FITs is not provided in CPRD, making this impossible to assess. However, guidelines recommending a referral cut-off of 10 µgHb/g faeces do not make any distinctions between analysers, making this irrelevant to clinical practice.^8^,^9^

## Conclusion

We have demonstrated a rapid increase across in England in the use of FIT since 2019 and that large differences exist between English regions in the use of FIT and the proportion meeting NICE criteria for secondary care referral for colorectal cancer investigation. Differences in patient behaviour or access to tests, the populations tested or the clinical pathways in place at primary care, secondary care or Integrated Care Board level within and between regions may explain these differences. A greater understanding of the reasons for the regional variation in FIT testing we have observed would contribute to efforts to reduce inequalities in FIT use, colorectal cancer diagnosis, treatment and survival.

## Data Availability

The data that support the findings of this study are available from CPRD. Restrictions apply to the availability of these data, which were used under license for this study. Data are available from the corresponding author (Alastair James Morton) with the permission of CPRD.
